# Can the Eight Hop Test Be Measured with Sensors? A Systematic Review

**DOI:** 10.3390/s22093582

**Published:** 2022-05-08

**Authors:** Luís Pimenta, Nuno M. Garcia, Eftim Zdravevski, Ivan Chorbev, Vladimir Trajkovik, Petre Lameski, Carlos Albuquerque, Ivan Miguel Pires

**Affiliations:** 1 Escola de Ciências e Tecnologia, University of Trás-os-Montes e Alto Douro, Quinta de Prados, 5001-801 Vila Real, Portugal; al70827@alunos.utad.pt; 2 Instituto de Telecomunicações, Universidade da Beira Interior, 6200-001 Covilhã, Portugal; ngarcia@di.ubi.pt; 3 Faculty of Computer Science and Engineering, University Ss Cyril and Methodius, 1000 Skopje, North Macedonia; eftim.zdravevski@finki.ukim.mk (E.Z.); ivan.chorbev@finki.ukim.mk (I.C.); trvlado@finki.ukim.mk (V.T.); petre.lameski@finki.ukim.mk (P.L.); 4 Health Sciences Research Unit: Nursing (UICISA: E), Nursing School of Coimbra (ESEnfC), 3046-851 Coimbra, Portugal; calbuquerque@essv.ipv.pt; 5 Higher School of Health, Polytechnic Institute of Viseu, 3504-510 Viseu, Portugal; 6 Child Studies Research Center (CIEC), University of Minho, 4710-057 Braga, Portugal

**Keywords:** Eight Hop Test, systematic review, measurement, sensors, diseases

## Abstract

Rehabilitation aims to increase the independence and physical function after injury, surgery, or other trauma, so that patients can recover to their previous ability as much as possible. To be able to measure the degree of recovery and impact of the treatment, various functional performance tests are used. The Eight Hop Test is a hop exercise that is directly linked to the rehabilitation of people suffering from tendon and ligament injuries on the lower limb. This paper presents a systematic review on the use of sensors for measuring functional movements during the execution of the Eight Hop Test, focusing primarily on the use of sensors, related diseases, and different methods implemented. Firstly, an automated search was performed on the publication databases: PubMed, Springer, ACM, IEEE Xplore, MDPI, and Elsevier. Secondly, the publications related to the Eight-Hop Test and sensors were filtered according to several search criteria and 15 papers were finally selected to be analyzed in detail. Our analysis found that the Eight Hop Test measurements can be performed with motion, force, and imaging sensors.

## 1. Introduction

The Eight Hop Test is a hop exercise that consists of jumping with one leg in a circuit in the form of the number eight [1,2]. This test is helpful to evaluate the physical strength of individuals that suffered from some disease related to the lower limb [2,3].

Different kinds of sensors available in the market allow the measurement of patterns related to different movements [4,5,6]. It can handle the creation of automatic methods to the empowerment of the physical treatments [7,8,9]. These methods are important to give the same opportunities to rural environments in terms of treatments and monitoring of health conditions [10,11,12]. There are different types of sensors, but the sensors that are especially important for these measurements are the sensors for motion detection, which are available in commonly used mobile devices [13,14,15]. The positioning of these devices has different limitations, but it is relatively easy due to different support straps that allow these devices’ statical position [16,17]. Therefore, the technology may help clinicians or scientists to study the detailed biomechanical parameters of jumping during rehabilitation programs as relevant variables for clinically significant scores and decide on the initiation of RTS with more confidence [18].

This review is included in a project related to the automation of the measurement of different results of the different physical functional tests, including the Heel-Rise Test [19,20], Timed-Up and Go Test [6,21], Ten Meter Walk Test [22], Six-Minute Walk Test [23], Functional Reach Test [24], 30-s Chair Stand Test [25,26], and Sit-to-Stand Test [27], where the development of different solutions involved in further studies. Furthermore, it is helpful and benefits the creation of Enhanced Living Environments [28,29]. The Eight Hop Test is mainly associated with different musculotendinous injuries, such as Cruciate Ligament of the Knee, Medial patellofemoral ligament, and Achilles tendon [18] and with injuries on the anterior cruciate ligament and gluteus medius [30,31].

The physical functional tests, such as bilateral or unilateral vertical and horizontal jump tests, require muscle strength and neuromuscular coordination for dynamic joint stability, which deteriorates with a knee injury [32,33]. In this regard, functional tests have been widely evaluated in laboratories using motion capture cameras, force platforms, and contact mattresses [32,34] to better understand biomechanical changes after knee injuries.

The monitoring of the biomechanics of the lower limbs during functional activities may help in the decision making related to the performance of sports or working activities after injury and prevention of osteoarthritis [35]. The analyzed test in this review allows the doctors to check the deficit of the significant underlying muscle deficits and ligament instability are still present throughout the post-operative rehabilitation period [35].

The study’s purpose consists of a systematic review related to the measurement of the results of the Eight Hop Test with sensors, including motion force and imaging sensors. With the Eight Hop Test, the sensors can reach different results.

This paragraph ends the introductory section of this systematic review. Next, Section 2 describes research questions, inclusion criteria, search strategy, and analyzed study characteristics. Section 3 shows each study’s results and summary. Third, in Section 4, we discuss and highlight the most critical points, and finally, Section 5 concludes the paper.

## 2. Methodology

### 2.1. Research Questions

The questions of this systematic review were focused on: (RQ1) Which devices can be used in the Eight Hop Test? (RQ2) Which data are related to the different types of diseases diagnosed by the Eight Hop Test? (RQ3) What are the benefits of implementing technological methods for measuring the results of the Eight Hop Tests?

### 2.2. Inclusion Criteria

The exercises and the sensors/equipment that had been used on the measurements of the Eight Hop Test results were based on the following inclusion criteria: (1) studies that measured different parameters related to the Eight Hop Test with sensors/equipment; (2) studies that used Eight Hop Test as the primary method; (3) studies that relate the Eight Hop Test with some diseases; (4) studies that clearly present results and population; (5) studies that were published between 2012 and 2021; (6) studies written in English.

### 2.3. Search Strategy

The Eight Hop Test consists of activities based on hopping used for rehabilitation purposes, and some studies only use a few of them. The search was performed with a Natural Language Processing (NLP)-based framework [36] in the following databases: IEEE Xplore; PMC; Pubmed Central; Springer; Association for Computing Machinery (ACM); Elsevier; and Multidisciplinary Digital Publishing Institute (MDPI). The keywords applied for this research were: “Eight hop test sensors”; “Eight hop test exercises”; and “hopping”. Each study was filtered using the defined criteria presented in Section 2.2. The research was performed on 2 November 2021.

### 2.4. Extraction of Study Characteristics

There are specific parameters extracted from the studies. The information from the studies was divided and presented in Table 1 by the following terms: year of publication; population; the purpose of the studies; sensor/equipment; types of methods; and diseases. Some studies do not mention all the information required in Table 1, but they always gave precious data to help answer the questions in Section 2.1. The lack of information provided in the studies was compensated by contacting the respective authors of the analyzed studies. The information related to implementing the Eight Hop Test with technological equipment is limited, and this subject needs more research.

## 3. Results

### 3.1. Summary of the Search Process Results

**Figure 1:**
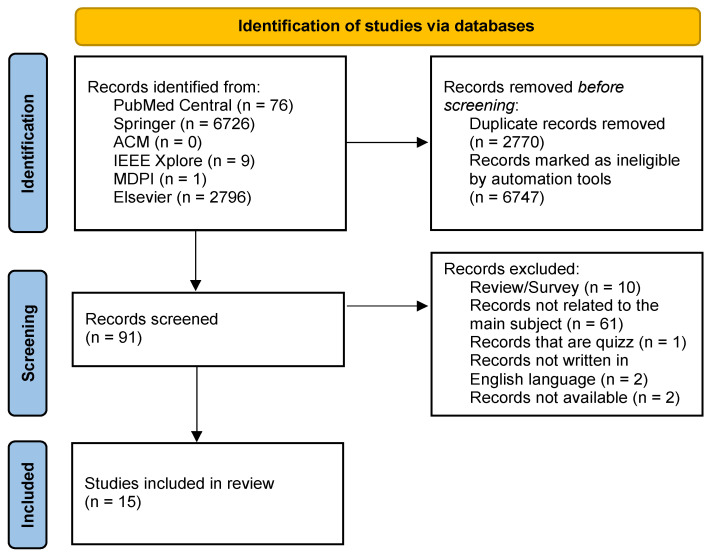
Flow diagram of identification and inclusion of papers.

As presented in Figure 1, the review has 9608 articles, 2770 of which are duplicates, and 6747 are marked as ineligible. For these reasons, the articles were removed correctly. The remaining 91 were filtered as well. In the filtering process (including the complete text evaluation), we found that 10 were Review/Survey, 61 were not related, one presented a quiz, two were not written in the English language, and two were not available. The remaining 15 papers were included in the qualitative synthesis and quantitative synthesis. In summary, we examined 15 scientific articles.

Based on the results presented in Table 1, the analyzed studies were published between 2012 and 2021, reporting that the major part of the studies was published in 2021 (five studies), and, before that, only 10 studies are scarcely distributed between 2012 and 2020. By analyzing the location where the studies have been performed, a major part of the studies were performed in the United States of America (four studies), Australia (three studies), and United Kingdom (two studies), where the remaining studies are distributed by the globe. Regarding the sensors/equipment used, the most relevant used are cameras (five studies), reflective markers (four studies), force plates (four studies), electrodes (three studies), accelerometer (three studies), stopwatches (two studies), ultrasound scanner (two studies), metronome (two studies), and measuring tape (two studies), where the remaining sensors/equipment are used in only one study. Regarding the different types of diseases, only nine studies included people with specific diseases, including injury in an anterior cruciate ligament (four studies), Achilles tendon injury (two studies), bronchiectasis (one study), gluteus medius injury (one study), and Patellofemoral ligament injury (one study). In addition, all the studies used statistical and mathematical methods to prove the test’s veracity.

### 3.2. Main Results, Benefits and Limitations of the Selected Studies

In Table 2, we summarize the main results, benefits and limitations of the selected studies relevant to measurement of the results of the Eight Hop Test.

### 3.3. Qualitative Synthesis of the Most Relevant Works

Baxter et al. [37] used four different types of sensors/equipment to implement several methods. The study used reflective markers, a 12-camera motion capture system, three-embedded force plates, and open-source musculoskeletal modeling software to perform the analysis. The study analyzes which rehabilitation exercises, such as single-leg hop, make a more robust and durable Achilles tendon. For that reason, eight young adults were put to the test. During the tests, enough data was collected to develop an exercise progression that helps increase the Achilles tendon’s strength based on the magnitude duration and rate of tendon loading. In conclusion, peak Achilles tendon loads varied more than 12-fold, from 0.5 bodyweights during a seated hell raise to 7.3 bodyweights during a forward single leg hop.

In [38], the authors used motion and force sensors, such as accelerometer and dynamometer, to study which Hop Tests syncs better with isokinetic knee extensor strength, the deficits after an injury in the anterior cruciate ligament reconstruction, and which hop test correlates more with isokinetic knee extensor strength. Thirty-four males and sixteen females with surgery in the past 9–12 months (16–50 years of age) were assessed for the study. The hop tests presented in the study are single (SHD), triple (THD), and triple crossover (TCHD) hop for distance, six minute timed hop (6 MTH), single medial (MHD), and single lateral (LHD) hop for distance, single countermovement jump (SLCMJ) and timed speedy hop (SHT). The results show that specific hop tests such as single medial and single countermovement jump correlated the most with isokinetic knee extensor when the more sophisticated testing equipment is lacking.

Ebert et al. [39] used an accelerometer and a stopwatch to find if the eight hop tests can identify limb asymmetry after anterior cruciate ligament reconstruction. For this study, fifty patients (34 males and 16 females) were assessed 9–12 months following anterior cruciate ligament reconstruction. The test was made in both limbs in a randomized order. These included single (SHD), triple (THD), and triple crossover (TCHD) hop for distance, six minute timed hop (6 MTH), single medial (MHD). Single lateral (LHD) hop for distance, single countermovement jump (SLCMJ), and timed speedy hop (TSHT). The results showed that single lateral hop, single medial hop, timed speedy hop, and single countermovement jump was the best physical exercises to demonstrate the functional limb asymmetry among the patients. In conclusion, if the purpose is to detect lingering functional deficits, it is recommended to incorporate the previous-hop test mentioned.

The authors of [40] tested the fundamental skill and physical activity of children with bronchiectasis using ActiGraph GT3x and an accelerometer to show if the performance is affected by the disease. Forty-six children with bronchiectasis (mean age 7.5 ± 2.6 years, 63% Male) were recruited from the Queensland Children’s Hospital, Brisbane. The children were measured by normal quotidian activities like sedentary, light-intensity, games, walking, running, and moderate-to-vigorous activities. The results showed that children with bronchiectasis tend to delay their fundamental skills development. Fewer than 5% of children demonstrated mastery in the run, gallop, hop, and leap, while fewer than 10% demonstrated the ability to perform the two-handed strike, overarm throw, and underarm throw. Only eight of the 46 children (17.4%) achieved their age equivalency for locomotor skills, while just four (8.7%) completed their object control skills. It is important to note that children in their age equivalency had significantly more time in daily physical activity during the tests.

The authors of [41] used SECA portable stadiometer, Nikon video camera, and Windows Media Player 2013 to examine primary school children’s fundamental movement skill proficiency levels. It recruited 219 participants (111 boys, 108 girls) aged between 7–10 years from three schools in central England to perform eight skills related to locomotor, object control, and stability skills. The eight fundamental skills involved run, jump, hop, skip, catch, overarm throw, underarm throw, and stability. The results find that any child could master all the fundamental skills mentioned. The conclusion says that to improve essential skills in all children, the effort should focus on stability skills (improving coordination) and force/power production.

In [42], the authors proposed an evaluation of a medial patellofemoral ligament using patient-reported measures and functional testing. For this study, 24 patients with a medical record between 2008 and 2013 were examined with a control group of uninjured persons of the same age and gender. The evaluation had two phases. In the first part, questionnaires evaluated the knee function based on the Tegner score, the knee injury and osteoarthritis outcome score (KOOS), the Lysholm score, SF-36, and EQ-5D-3L. The second part was the functional performance that involved square jump, steps down test, and the single-leg hop for distance. The results were: patients 11.5 sets for the square jump versus control 21 sets, 11.5 sets for the step-down test versus control 22 sets, and 77 cm for the single-leg hop for distance versus control 126 cm. The patients showed worse results than the control group in all tests, which led the study to conclude that patients with a medial patellofemoral ligament reconstruction do not regain normal knee function.

In [43], the authors examine the functional outcomes, including static-dynamic postural stability of patients with an associated gluteus medius treated injury. For this study, 16 patients were chosen with the clinical record (treated with an antegrade trochanteric IMN) between January 2009 and July 2013 and eight healthy male controls. Some data was gathered before the physical activity, including muscle strength, static and dynamic postural stability, and fall risk. The measurements included the participation of imaging sensors electromyography (EMG). The study results showed that patients with an antegrade trochanteric IMN are more likely to have a good balance but poor functional performance. Still, more studies are needed to find the reason behind the results.

In [44], the author did not use any sensor to perform the test, which was evaluated by an examinator. The study considered 16 non-injured participants and 32 anterior cruciate ligament reconstruction participants. It was intended to examine the test-retest reliability of single-hop tests in the forward, medial and rotational direction and then detect limb asymmetries of the medial rotational hop tests, compared to forward hop tests made for the participants with a reconstructed anterior cruciate ligament. For the tests, they used some hop exercises like the single hop for distance (SH), triple hop for distance (TH), medial side triple hop for distance (MSTH), and 90° medial rotation hop for distance (MRH). The non-injured participants were tested twice, and the anterior cruciate ligament participants once. To prove the methods, it was calculated the intraclass correlation coefficients (ICCs), the standard errors of measurement (SEM), and the most negligible detectable differences (SDD). In the end, these exercises are reliable for rehabilitation purposes. Medial and rotational hope tests have the probability of showing limb asymmetries in a person with a reconstructed anterior cruciate ligament compared to the forward hope test.

In [45], the study’s authors examine the best exercises (including hopping) in runners with Achilles tendinopathy based on self-reported pain. Fifteen male runners with Achilles tendinopathy were tested by loading the Achilles tendon with running, sprinting, hopping, jumping, and morning stiffness. The pain was measured before and after the workout with a numeric rating scale where zero means “no pain” and ten is “the worst possible pain”. In total, 100% of the participants were recruited, 87% retention, and 93% followed-up. Exercise adherence was 70%. However, fidelity was 50%. Three participants suffered adverse events due to not following the advised exercises. Still, five participants were satisfied, and eight were very satisfied. In conclusion, the recommended education and training with pain-guided hopping positively impacts recreational runners with Achilles Tendinopathy.

Owusu-Akyaw et al. [46] used a magnetic resonance scanner to extract images from the knees before and after each subject performed a series of 60 single-legged hops. Then the images were converted into three-dimensional surface models of cartilage and bone to assess the cartilage characteristics in terms of thickness distribution. Eight male subjects with unilateral anterior cruciate ligament consented to participate in this study. The results found that the anterior cruciate ligament was associated with decreased patellar cartilage thickness by noticing that exercise would induce cartilage strain compared to the uninjured knees.

Reuter et al. [47] took the top German decathlon team, a group of eight professional athletes, to perform some high-end exercises to study postural control while exercising. Star Excursion Balance Test (SEBT), single hop for distance (SLH), crossover hop for distance (COH), triple hop for distance (TH) were used to perform the studies. The results demonstrated a correlation between the single-leg hop test and the star excursion balance test in terms of performance. These two exercises are the most efficient to determine overall postural control in athletes. For measurements, a measuring tape was used.

The author of [48] used Wireless electrodes, ultrasound probe, athletic tape, retro-reflective markers, and MX03 + NIR Cameras to perform the studies in eight college-aged males with no musculoskeletal injury. The study’s primary purpose was to investigate changes in plantar flexor contractile component length, plantar flexor muscle activity, and tendon length and how it could reduce mechanical efficiency during exhaustive hopping exercises. For the study, eight college-aged males with no musculoskeletal injury, neuromuscular disease, or functional limitation in their legs participated in a complete hopping exercise to the absolute limit. In that time, the data was collected and analyzed. The results found that the mechanical efficiency of hopping did not change and remained the same.

Wibawa et al. [49] used a Gait Laboratory (Dept of Rehabilitation Medicine, UMCG, The Netherlands) to perform the tests, including imaging and force sensors to analyze muscle activities like normal walking, one-legged forward, and side jumping with a Musculoskeletal Modeling System. A nine-meter-long walkway, force plates, Vicon Motion System, cameras, reflective markers, and electrodes were used to measure and analyze ten healthy subjects (six males and four females) during the exercises. Each subject was evaluated, and then the values obtained by doing muscle activity were compared with the Musculoskeletal Modeling System. Some individuals were excluded during the study due to abnormal walking, marker trajectory error, and errors in market data. The other included three trials contributing enough data to conclude the investigation. The electrodes measured the right leg of the subjects. The correlation between sensors and the Eight Hop Test can be a game-changing move when the problem is finding how the injuries comport during the exercises. In conclusion, the study showed differences between the data and the model extracted from the Musculoskeletal Modeling System.

In [50], the authors used force plates, cameras, retro-reflective markers, and a digital metronome to analyze the center of pressure locations during two-legged hopping. By following the university ethics committee’s approval, eight healthy and active adults (five females; three males) consented to participate in the study, doing at least ten jumps in a specific frequency. The attachment made the measurements of retro-reflective markers to a particular joint (metatarsophalangeal joint). The results showed that using retro-reflective markers in specific joints can determine the center of pressure during quiet standing and two-legged hopping at a particular frequency. Still, the results are limited to quiet standing and two-legged hopping in healthy adults. For that reason, more investigation is required to assure the accuracy of the method in walking and running or with clinical populations.

The authors of [51] used a population of fifteen college-age males, with right lower extremity dominance, to determine which exercises related to strength, endurance, flexibility, motor control, and function are more reliable in clinical measurements. It used Biodex System 3 pro and Biodex Balance System SD to collect some data before their studies began. For each test, there was a different exercise. Strength: eight isometric tests and a sit-up test. Endurance tests: the trunk flexor test, trunk extensor test, and bilateral side bridge tests. Flexibility tests: the sit-and-reach test and active range of the trunk and hip joint motions. Motor control: limb balance test and proprioception via passive reposition tests of the hips. Functional: squat test and single-leg hop test for time and distance. The results showed that endurance tests are the most reliable for clinical measurements, followed by flexibility, strength, motor control, and functionality.

### 3.4. Relationship between Studies, Sensors and Diseases

For this section, Table 3 represents a relation between sensors and diseases used to prove that studying different types of diseases directly related to the Eight Hop test is important. Still, the combination of the different methods and well-formed strategies has equal importance when monitoring people with various diseases using the Eight Hop test.

## 4. Discussion

### 4.1. Summary of Relationship between Sensors and Diseases

The Eight Hop Test, specifically, was not present in any studies. However, the data collected from each study can help us understand which sensors are used in Hop Tests since the Eight Hop Test is a part of Hop Tests.

**Figure 2:**
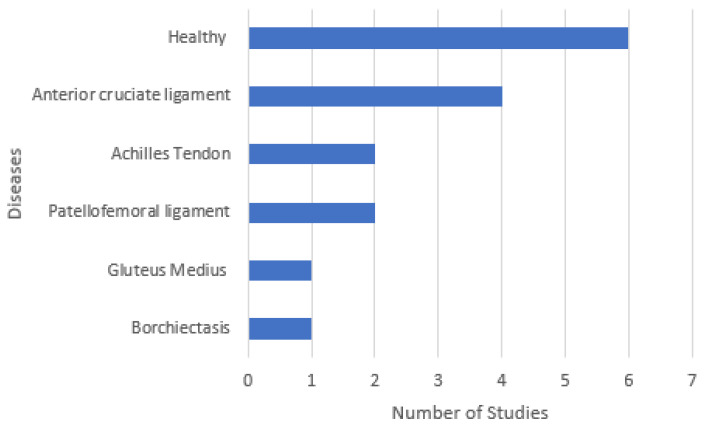
Distribution of the various diseases by the studies.

Some studies were based on problems related to a specific disease, and Figure 2 demonstrates various diseases that conducted the studies, where six studies were made with healthy people, four studies were performed with people with anterior cruciate ligament reconstruction, and the other studies included people with Achilles tendon (two studies) and patellofemoral ligament (two studies) as the leading injury. Gluteus Medius and Borchiectasis were mentioned in one publication each.

As presented in Table 4, the sensors available in the different studies were distributed in various categories, such as medical sensors, motion sensors, time counting equipment, imaging sensors, force sensors, and support equipment/consumables. Regarding the sensors used in the various studies, force sensors (four studies), such as force plates, dynamometer, Biodex System 3 pro, and Biodex Balance System SD had the most variety with four different sensors, followed by imaging sensors (seven studies), such as cameras, magnetic resonance scanner, and ultrasound scanner, and motion sensors (three studies), such as accelerometer, Vicon motion system, and Actigraph GT3x that had a variety of three different sensors.

### 4.2. Relationship between Ages of Participants and Studies

**Figure 3:**
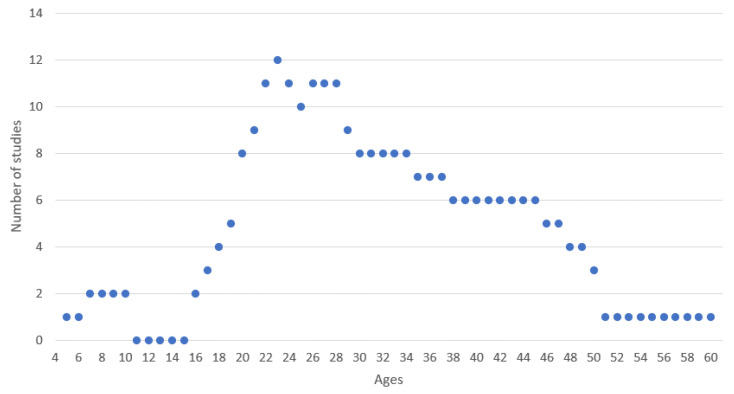
Relation of ages and number of studies.

Regarding people’s gender, the studies averaged 20.6 males and 12.8 females, including children and adults. Figure 3 presents the distribution of the ages of the different participants in the analyzed studies, where more than seven studies included individuals aged between 20 and 34 years old.

All studies used statistical and mathematical methods to study the results. The most used feature was the distance followed by time and the number of repetitions. Not all studies used sensors as the primary source of collecting data, where some of them were based on measuring distances and examining motion captures.

### 4.3. Final Remarks

We can conclude that more studies are needed to develop a global solution for precise measurements. There is no proven evidence that the use of sensors in the Eight Hop test is essential, but according to the studies, it helps contribute to the fidelity and viability of the measurements.

After a deep analysis of the fifteen studies presented in this systematic review, we can find answers to our main questions. Regarding the RQ1, “Which devices can be used to perform studies in the Eight Hop Test?”, we verified that the most common sensors used were the imaging sensors such as cameras, magnetic resonance, and ultrasound scanners. Furthermore, we have force sensors, time counting sensors, and motion sensors. More than half of the studies mention the need for support equipment/consumables, helping in the measuring, and completing the purpose of the sensors in these studies.

Concerning RQ2, “Which data are related to the different types of diseases diagnosed by the Eight Hop Test?”, the analyzed studies show that different diseases require different sets of sensors and sensor data. For the same disease, for example Anterior cruciate ligament, different studies use different sensors. Additional research is needed to find out which sensor gives the best results, since no comparative analysis could be performed due to the varying experimental setups of the analyzed studies. Table 3 shows the relation between the sensors and the various diseases.

Finally, regarding RQ3, “What are the benefits of implementing technological methods for the measurements of the results of the Eight Hop Tests?”, we verified that in addition to the limitations presented in Table 2 the studies showed some benefits related to rehabilitation and empowerment on the clinical information.

More studies are needed on this topic, but one thing is sure: we can use sensors to measure possible results prevenient from the Eight Hop Test.

## 5. Conclusions

In this review, a total of 15 studies were selected based on the inclusion criteria and thoroughly analyzed. The review identified which sensors are used in the Eight Hop Test, which are the most used sensors, the relevance of sensors in measurements, which diseases are related to the Eight Hop Test, and which methods can be used to perform the Eight Hop Test. It is important to mention that there is a lack of studies to develop a method for analyzing the Eight Hop Test with sensors. However, the sensors increase the viability of the measurements and help clinical teams to perform better diagnostics in health.

As future work, a mobile application will be developed to create a new method for the commodity measurement of the results of the Eight Hop Test that will be integrated with other ongoing studies related to the construction of a Personal Digital Life Coach.

## Figures and Tables

**Table 1:** Study analysis.

Paper	Year of Publication	Location	Population	Purpose of the Study	Sensors/Equiment	Diseases Studied
Baxter et al. [37]	2021	United States of America	10 healthy adults	The study analyses which Hop exercises makes a stronger and durable Achilles tendon	Reflective marker; 12 camera motion capture system; Force plate	Achilles tendon fracture
Ebert et al. [38]	2021	Australia	34 males and 16 females	The study focus on which Hop Tests syncs better with isokinetic knee extensor strength and the deficits after an injury in the anterior cruciate ligament reconstruction	Stopwatch; Accelerometer; Velcro strap; Isokinetic dynamometer	Anterior cruciate ligament
Ebert et al. [39]	2021	Australia	34 males and 16 females	This study aimed to find if the eight hop tests can identify limb asymmetry after anterior cruciate ligament reconstruction	Accelerometer; Stopwatch	Anterior cruciate ligament
Joschtel et al. [40]	2021	Australia	46 children	Comparison fundamental movement skill proficiency in children with bronchiectasis with measured Physical Ability	ActiGraph GT3X; Accelerometer	Bronchiectasis
Lawson et al. [41]	2021	United Kingdom	111 males and 108 females	Studying the fundamental movement skill levels in primary school children	SECA portable stadiometer; Nikon video camera;	Healthy
Biesert et al. [42]	2020	Sweden	24 patients	This study proposed an evaluation of a medial patellofemoral ligament using patient reported measures and functional testing	Goniometer	Patellofemoral ligament
Ergişi et al. [43]	2020	Turkey	15 males, 1 female and 8 healthy male controls	This study examines the functional outcomes, static-dynamic postural stability of patients with an associated gluteus medius treated injury	Wireless electromyography; Bipolar adhesive surface electrodes	Gluteus medius
Dingenen et al. [44]	2019	Belgium	16 non-injured participants & 28 anterior cruciate ligament reconstructed participants	The study had 2 purposes. The first one was to examine the test-retest reliability of single hop tests in the forward, medial and rotational direction. The second one was to detect limb asymmetries of the medial rotational hop tests in comparison with forward hop tests	Measuring tape	Anterior cruciate ligament
Sancho et al. [45]	2019	United Kingdom	15 male recreational runners	The study examines the best hopping exercises in runners with Achilles tendinopathy based on a self-reported pain	Metronome; Ultrasound scanner	Achilles tendinopathy
Owusu-Akyaw et al. [46]	2018	United States of America	8 male subjects	Comparison between anterior cruciate ligament deficient and intact knees in subjects due to patellofemoral joint and mechanics	Magnetic Resonance (MR) scanner	Anterior cruciate ligament; Patellofemoral joint osteoarthritis
Reuter et al. [47]	2017	Germany	8 professional athletes	This study proposed to show a relation of different dynamic postural control tests in healthy professional athletes and their measures	Measuring tape	Healthy
Lidstone et al. [48]	2016	United States of America	8 college-aged males	This study investigates changes in plantar flexor contractile component length, changes in plantar flexor muscle activity and tendon length and how it could reduce mechanical efficiency during exhaustive stretch-shortening cycle exercises	Wireless electrode; Ultrasound scanner; Athletic Tape; Retro-reflective markers; MX03 + NIR Cameras	Healthy
Wibawa et al. [49]	2016	Indonesia	10 healthy subjects	Analyses muscle activities like normal walking, one-legged forward and side jumping with a Musculoskeletal Modeling System	9 m long walkway; force plates; Vicon Motion System; Ten cameras; Reflective markers; Electrodes	Healthy
Furlong et al. [50]	2014	Ireland	7 healthy active adults	Analyses the center of pressure locations during two-legged hopping	Cameras; Force Plates; metronome; Retro-reflective markers	Healthy
Waldhelm et al. [51]	2012	United States of America	15 college-age males	This study determines which exercises related to strength, endurance, flexibility, motor control and function are more reliable in clinical measurements	Biodex System 3 pro; Biodex Balance System SD	Healthy

**Table 2:** Study results and benefits.

Study	Results and Benefits	Limitations
Baxter et al. [37]	The results gave enough data to develop a method to measure exercise progression that helps increase the Achilles tendon’s strength based on the magnitude duration and rate of tendon loading	Only eight healthy adults were included in the study, the population is limited. Test were made only on healthy people that contradicts the purpose of the study (rehabilitation)
Ebert et al. [38]	The results show that specific hop tests such as single medial and single countermovement jump correlated the most with isokinetic knee extensor when the more sophisticated testing equipment is missing. The hop measurements of study can inform the clinician of the possible existence of significant underlying quadriceps deficits are still present even after the operative rehabilitation period.	N/D
Ebert et al. [39]	The final results showed that single lateral hop, single medial hop, timed speedy hop, and single countermovement jump were the best physical exercises to demonstrate the functional limb asymmetry among the patients.	N/D
Joschtel et al. [40]	Results showed that children who suffer from bronchiectasis are more likely to not achieve age equivalency for locomotor skills and for object control skills. However, there were no differences for sedentary activities, light-intensity activities and games, waling, and running.	The children who met their age equivalency for fundamental skill had more time spent in daily physical activity than the other who did not.
Lawson et al. [41]	The results find that any child could master all the fundamental skills mentioned. However, the study gave precious knowledge, it was found that to improve essential skills in all children, the effort should focus on stability skills and force/power production.	N/D
Biesert et al. [42]	The results showed that patients had worse results than the control group in all tests, which led the study to conclude that patients with a medial patellofemoral ligament reconstruction do not regain normal knee function.	N/D
Ergişi et al. [43]	The study results showed that patients with an antegrade trochanteric are more likely to have a good balance but poor functional performance.	The results cannot be explained by the study, further studies are needed.
Dingenen et al. [44]	Results showed that medial and rotational hope tests have the probability of showing limb asymmetries in a person with anterior cruciate ligament reconstructed compared to the forward hope test.	There are no sensors included in the study, for the tests, a rolling tape was used. The uninjured were only tested twice and the ACL-reconstructed participants once.
Sancho et al. [45]	The results showed that education and training with pain-guided hopping has positive impacts in recreational runners with Achilles Tendinopathy	Parts of the results were justified by the participants. Three participants did not follow up the advised activities.
Owusu-Akyaw et al. [46]	The results found that the anterior cruciate ligament was associated with decreased patellar cartilage thickness by noticing that exercise would induce cartilage strain compared to the uninjured knees	The first limitation is the fact that only eight subjects were used for the study. Second, they were all male, that excludes a comparison with female subjects.
Reuter et al. [47]	Results demonstrated a correlation between the single-leg hop test and the star excursion balance test in terms of performance. These two exercises are the most efficient to determine overall postural control in athletes	The population of the study was formed by male athletes only, that excludes a possible comparison with female athletes.
Lidstone et al. [48]	The results found that the mechanical efficiency of hopping did not change and remained the same.	The population of the study was formed by male participants only, which excluded a possible comparison with female participants.
Wibawa et al. [49]	Results showed that the study can be used as baseline for scientific work, to get more reliable and robust musculoskeletal models, as it contributes to an uncertainty reduction.	The first limitation is that six subjects had to be excluded due to abnormal walking, marker trajectory errors, and errors in marker data. That leads to a small population. Second, the Modeling software can possibly miscalculate the knee net moment, absence of co-contraction, and simplified knee joint.
Furlong et al. [50]	The results showed that using retro-reflective markers in specific joints can determine the center of pressure during quiet standing and two-legged hopping at a particular frequency.	The results are limited to quiet standing and two-legged hopping in healthy adults. For that reason, more investigation is required to assure the accuracy of the method in walking and running or with clinical populations.
Waldhelm et al. [51]	Results showed that endurance tests are the most reliable for clinical measurements, followed by flexibility, strength, motor control, and functional.	The population of the study was formed by male participants only, which excludes a possible comparison with female participants.

**Table 3:** Relation between diseases and sensors used.

Study	Sensors Categories						Diseases					
	Medical Sensors	Motion Sensors	Time Counting Sensors	Imaging Sensors	Force Sensors	Support Equipment/Consumables	Anterior cruciate ligament	Healthy	Bronchiectasis	Achilles Tendon	Gluteus Medius	Patellofemoral ligament
Baxter et al. [37]				X	X	X				X		
Ebert et al. [38]		X	X		X		X					
Ebert et al. [39]		X	X				X					
Joschtel et al. [40]		X							X			
Lawson et al. [41]				X		X		X				
Biesert et al. [42]						X						X
Ergişi et al. [43]	X										X	
Dingenen et al. [44]						X	X					
Sancho et al. [45]			X	X						X		
Owusu-Akyaw et al. [46]				X			X					X
Reuter et al. [47]						X		X				
Lidstone et al. [48]				X		X		X				
Wibawa et al. [49]				X	X	X		X				
Furlong et al. [50]			X	X		X		X				
Waldhelm et al. [51]					X			X				

**Table 4:** Relation between sensors used and its categories.

Sensors Categories	Sensors
Medical sensors	Electromyography
Motion Sensors	Accelerometer
	Vicon Motion System
	Actigraph GT3x
Time counting equipment	Stopwatches
	Metronome
Imaging sensors	Cameras
	Magnetic Resonance scanner
	Ultrasound scanner
Force sensors	Force Plates
	Dynamometer
	Biodex System 3 pro
	Biodex Balance System SD
Support equipment/consumables	Reflective markers
	Electrodes
	9-m long walkway
	Athletic tape
	Measuring tape
	Velcro strap
	Stadiometer
	Goniometer

## Data Availability

Not applicable.
